# Developmental cues for the maturation of metabolic, electrophysiological and calcium handling properties of human pluripotent stem cell-derived cardiomyocytes

**DOI:** 10.1186/scrt406

**Published:** 2014-01-28

**Authors:** Wendy Keung, Kenneth R Boheler, Ronald A Li

**Affiliations:** 1 Stem Cell and Regenerative Medicine Consortium, LKS Faculty of Medicine, University of Hong Kong, Hong Kong; 2 Department of Physiology, University of Hong Kong, Hong Kong; 3 Division of Cardiology, Johns Hopkins University School of Medicine, Baltimore, MD 21205, USA; 4 Cardiovascular Research Center, Icahn School of Medicine at Mount Sinai, One Gustave L. Levy Place, New York, NY 10029, USA

## Abstract

Human pluripotent stem cells (hPSCs), including embryonic and induced pluripotent stem cells, are abundant sources of cardiomyocytes (CMs) for cell replacement therapy and other applications such as disease modeling, drug discovery and cardiotoxicity screening. However, hPSC-derived CMs display immature structural, electrophysiological, calcium-handling and metabolic properties. Here, we review various biological as well as physical and topographical cues that are known to associate with the development of native CMs *in vivo* to gain insights into the development of strategies for facilitated maturation of hPSC-CMs.

## Introduction

Despite advances in treatment, cardiovascular diseases continue to be the leading cause of death worldwide. Owing to the non-regenerative nature of terminally differentiated cardiomyocytes (CMs), myocardial repair remains severely limited by the source of viable CMs for replacement. Human pluripotent stem cells (hPSCs), including human embryonic stem cells (hESCs) and induced pluripotent stem cells (iPSCs), can propagate indefinitely while maintaining their ability to differentiate into virtually all cell types, including CMs. As such, hESCs/iPSCs provide an unlimited *ex vivo* source of CMs for clinical application and other purposes, such as drug discovery and cardiotoxicity screening. Whereas efforts have been made to develop highly efficient protocols for deriving hPSC-CMs, it is now widely accepted that their functional and structural properties are immature in multiple aspects, with embryonic- or fetal-like electrophysiological, calcium-handling and metabolic signatures. Here, we review recent efforts that have been made to understand the different biological cues for driving maturation.

## Directed cardiac differentiation of human embryonic stem cells/induced pluripotent stem cells

The first protocol of directed cardiac differentiation involves the co-culture of hESCs with mouse visceral endoderm-like cells (END-2) [1]. Subsequently, two methods involving embryoid body (EB) formation or monolayer culture have been developed. The EB method involves formation of spherical cell aggregates [2] that produce cell types from all three germ layers. Early protocols depend on formation of spontaneous contraction of the EBs, which has an efficiency ranging from 5 to 15%. Differentiation efficiency can be achieved by replacing serum-containing medium with growth factors and small chemical compounds in defined medium. Varying factors such as fetal bovine serum and insulin free medium, mitogen-activated protein kinase inhibitors [3], ascorbic acid [4] and insulin-like growth factors 1 and 2 [5] has been shown to enhance cardiac progenitor cell proliferation or CM proliferation. An improved protocol from Keller’s group, involving addition of low bone morphogenetic protein (BMP)4 levels during EB formation and the subsequent use of fibroblast growth factor 2, activin A, vascular endothelial growth factor A and dickkopf homolog 1, yields 70% of EBs with spontaneous contraction [6]. Other variants of this protocol involve addition of small molecule inhibitors of WNT signaling during later stages [7]. More developed versions that rely on EB formation have shown greatly increased differentiation efficiency to approximately 94% spontaneously beating EBs in a number of hESC and human iPSC lines [8]. In an improved version of this EB formation protocol, addition of the small molecule WNT inhibitor IWR-1 at day 4 yields over 90% CMs at day 15, with the appearance of beating clusters as early as day 8 [9].

Besides EB formation, a monolayer method has been developed with beating cells appearing 12 days post-differentiation. Laflamme and colleagues [10] developed a method where hESCs are cultured to a high confluency and treated with high concentrations of activin A followed by BMP4. Secreted factors are then allowed to accumulate for 4 days and contracting cells can be seen at day 12 with approximately 30% CMs. Improvements to this protocol involved the addition of WNT3A at days 0 to 1 and DKK at days 5 to 11, which improved the yield of CMs [11]. As with EB formation, addition of small molecule WNT inhibitors including IWR-1 and IWP-4 at day 3 has proven successful [12].

Our laboratory has recently developed a highly cost-effective and efficient system for deriving hPSC-CMs from hESC (HES2, H7, H9) and iPSC lines [13]. This protocol, based on EB formation, requires minimal reagents (no basic fibroblast growth factor and vascular endothelial growth factor required) to allow cardiac differentiation with a high efficiency for different hPSC lines. Early addition of activin A and BMP4 and addition of Wnt inhibitor at a later time point with ascorbic acid are sufficient to trigger CM differentiation among hESC and human iPSC lines with no need for titration of growth factors to achieve high efficiency CM differentiation in various hPSC lines. A final output of 35 to 70 ventricular hPSC-CMs per hPSC initially seeded for culture can be achieved, and hESC-CMs are capable of spontaneous beating starting at day 8 after initiation of differentiation. This simplified protocol may be easily adapted for mass production of ventricular hPSC-CMs in bioreactors.

## Human pluripotent stem cell-derived cardiomyocytes are structurally and functionally immature

Studies using various methods of cardiac differentiation show that hESC-derived CMs are immature and display fetal-like, and sometimes embryonic-like, properties [14]. Maturation of hESC-CMs is affected by cultivation time and culture conditions as well as co-culture with other cell types [15]. However, the effect of these modifications on maturation remains limited and the exact mechanisms and factors affecting maturation are still largely unknown.

**Figure 1:**
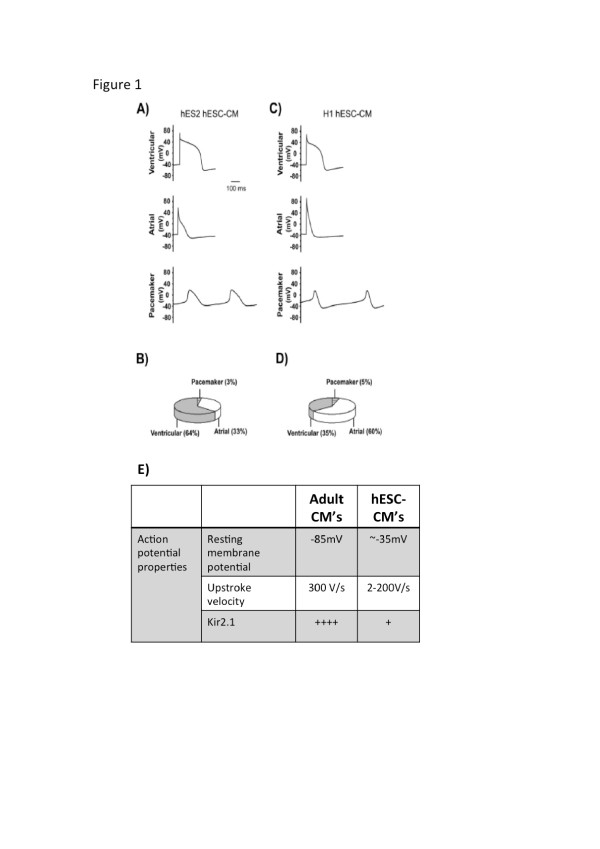
**Characteristics of action potentials in human embryonic stem cell-derived cardiomyocytes. (A-D)** Action potentials and percentage distribution of ventricular, atrial and pacemaker cardiomyocytes derived from HES2 **(A,B)** and H1 **(C,D)** human embryonic stem cells (hESCs). **(E)** Summary of differences in action potential properties between adult cardiomyocytes (CM) and hESC-CMs. (Adapted from Moore *et al*. [19]).

hESC-CMs display embryonic- or fetal-like structures. While adult human CMs are rod shaped with lengths in the 100 μm range, hESC-CMs are smaller in size (10 to 20 μm in diameter) and often round [16]. These cells tend to increase in size with prolonged time in culture; however, the shape of these cells remains round or oblong [14,16]. In terms of the contractile machinery, hESC-CMs show poor contractile protein organization with very low myofibrillar density as shown by sarcomeric α-actinin staining. Myofibrils in these cells have random structures within the cytoplasm, with no discernable A, I and Z bands [17]. Instead, immature Z-bodies joining clusters of adjacent sarcomeres are often found in hESC-CMs [14,17]. Sarcomeric length is also considerably shorter than that found in adult CMs. Although contractile machinery organization tends to improve with long-term culture, hESC-CMs continue to display no t-tubule [16,17] or M band formation [17], indicating that they cannot reach a level of maturity comparable to that of functional adult CMs. While adult CMs tend to be multinucleated, hESC-CMs are mononucleated [15]. With long-term culture, hESC-CMs tend to develop multinucleation at a percentage that is comparable to adult CMs [17]. However, engineered fusion of hESC-CMs does not lead to more mature electrophysiological or calcium-handling phenotypes (CW Kong and RAL, unpublished data). Depending on the culturing conditions and the cell line they are derived from, hESC-CMs may display different rates of beating, ranging from 30 to 80 beats/minute [18]. CMs derived from hPSCs display atrial-, ventricular- and pacemaker-like electrophysiological properties, with cardiogenic preferences that are dependent on the different hESC lines. For example, HES2 cells have been reported to be more likely to differentiate into ventricular CMs than H1 cells [19] (Figure 1).

Mature adult CMs are electrically quiescent but excitable upon stimulation. hESC-CMs display a greater degree of automaticity, with the remaining quiescent cells being able to elicit single action potentials upon stimulation [20]. Several studies have demonstrated the immature nature of the action potential in hESC-CMs (summarized in Figure 1E). Maximum diastolic potential is depolarized at around -30 mV to -58 mV in early hESC-CMs and becomes hyperpolarized to approximately -70 mV in late hESC-CMs [17,21-24]. This is, however, still more positive than the -80 mV normally seen in adult CMs. The upstroke velocity, which is about 300 V/s in adult CMs [25], ranges from 2 V/s to >200 V/s in hESC-CMs, which is slower than their adult counterparts by two to three orders of magnitude [17,22,26]. The action potential profile in hESC-CMs is, in general, immature and similar to that of arrhythmogenic, failing adult ventricular CMs with a prominent ‘phase 4-like’ depolarization and a significantly depolarized resting membrane potential [20]. Stimulation of hESC-CMs with the β-agonist isoproterenol results in increased contraction rate, increased amplitude of the calcium transient, and decreased relaxation time [27]. Unlike with adult CMs, however, increasing isoproterenol concentration produces no ionotropic response [28,29], once again demonstrating the immaturity of these cells.

**Figure 2:**
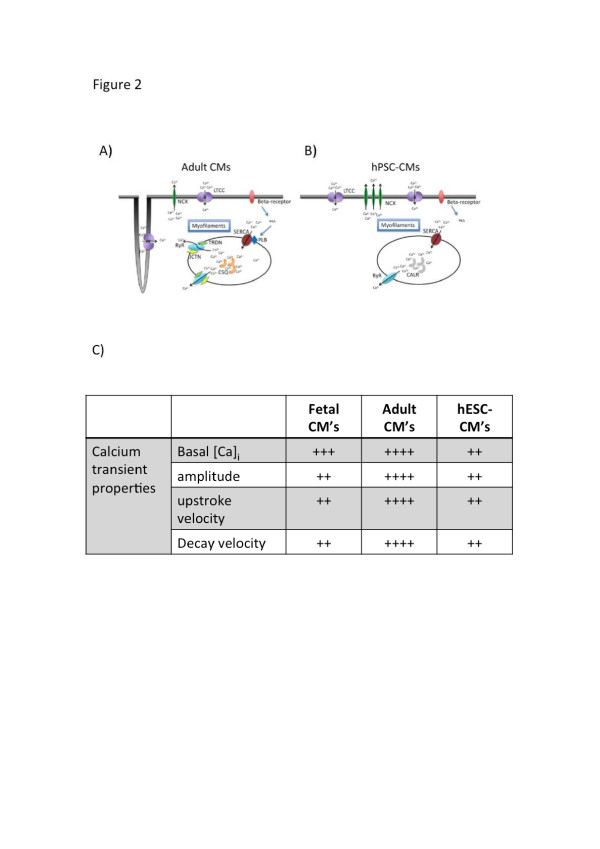
**Calcium handling properties in human embryonic stem cell-derived cardiomyocytes. (A,B)** Schematic showing calcium signaling pathways in adult cardiomyocytes (CM) **(A)** and human pluripotent stem cell-derived CMs (hPSC-CMs). hPSC-CMs show a smaller calcium transient amplitude, slower kinetics and absence of inotropic responses compared to adult CMs due to 1) lack of junctin and triadin to facilitate ryanodine receptor (RyR) function; 2) lack of calsequestrin for sarcoplasmic reticulum (SR) calcium buffering; 3) lack of phospholamban for sarco/endoplasmic reticulum Ca^2+^ ATPase (SERCA) modulation; 4) lower SERCA and RyR expression; 5) lack of T-tubules leading to U-shape of calcium propagation wavefront. (Adapted from Li *et al*. [33]). **(C)** Summary of differences in calcium transient properties between adult CMs and human embryonic stem cell-derived CMs (hESC-CMs). (Adapted from Kong *et al*. [34]).

In mature adult CMs, membrane depolarization during an action potential leads to the opening of sarcolemmal voltage gated L-type calcium channels, which are located at the T-tubular network lining the sarcolemmal membrane in close proximity to the sarcoplasmic reticulum (SR). Calcium entrance through the L-type calcium channels in turn triggers the rapid release of calcium from the SR via ryanodine receptors (RyRs) through a mechanism known as calcium-induced calcium release [30]. This in turn leads to a uniform increase in cytosolic calcium, which binds to troponin. Calcium binding causes a change in the shape of troponin that causes tropomyosin to shift its position along the actin filament, thus permitting myofilament contraction between actin and myosin. While adult CMs show a positive force-frequency relationship when paced, a negative force-frequency relationship is observed in hESC-CMs [20,31] (refer to Figure 2 for comparison of calcium handling between adult CMs and hESC-CMs). This suggests that hESC-CMs possess little SR function and t-tubules, and rely mostly on trans-sarcolemmal calcium influx, which slowly enters the cytoplasm, to increase intracellular calcium [16]. Reports on the degree of maturation of the SR in hESC-CMs vary. Early studies reported that cytosolic calcium transients in hESC-CMs do not respond to caffeine or ryanodine, suggesting that the SR in hESC-CMs is underdeveloped or non-functional, and that most contraction in hESC-CMs results from trans-sarcolemmal calcium influx rather than calcium release from the SR [31]. More recent studies from our laboratory [32] and others show that functional SRs are present even in young hESC-CMs, which, upon electrical stimulation, could generate Ca^2+^ transients similar to fetal left ventricular CMs. The amplitude of the upstroke and decay velocity in hESC-CMs also increases in long-term culture [17]. However, caffeine-induced Ca^2+^ release was observed in only a small percentage of hESC-CMs (40% of H1- and HES2-CMs versus 60% in fetal ventricular CMs). Ryanodine significantly reduced the electrically evoked Ca^2+^ transient amplitudes and slowed the upstroke of caffeine-responsive hESC-CMs. By measuring thapsigargin- and tetracaine-sensitive Ca^2+^ sparks as the fundamental events of Ca^2+^ handling, we directly demonstrated that calcium-induced calcium release is indeed functional in hPSC-CMs [33].

Besides having an underdeveloped SR, hESC-CMs show a pattern of expression of key calcium handling proteins that differs from that of adult CMs. While in adult CMs excitation-contraction coupling is mediated mainly by calcium-induced calcium release, in hESC-CMs it is primarily due to trans-sarcolemmal influx of calcium. Calcium transients in hESC-CMs have been shown to be dependent on L-type calcium channels, which can be blocked more than 80% by the specific L-type channel blocker nifedipine [35]. The residual calcium transient elicited after the blockade of nifedipine is facilitated by the sodium calcium ion exchanger NCX. As in fetal relative to adult CMs, hESC-CMs have been shown to have an increased expression of NCX, which works in the reverse mode to contribute to the calcium transient [20,35]. Our laboratory shows that NCX does not contribute to the calcium transient in ventricular hESC-CMs as indicated by the lack of effect of NCX inhibitors as well as its downregulation by short hairpin RNA [33]. Other calcium handling proteins normally present in adult CMs, including calsequestrin and phospholamban, have been shown to be absent in hESC-CMs [31,36], although there are reports that they are expressed in hESC-CMs [37,38]. The expression of sarco/endoplasmic reticulum Ca^2+^ ATPase (SERCA) pump in hESC-CMs is low and comparable to the levels in fetal CMs. However, only caffeine-sensitive CMs show a decrease in the decay of the calcium transient when SERCA is inhibited by thapsigargin in hESC-CMs, suggesting that maturation of SERCA is incomplete [32].

## Immature bioenergetics and metabolism in human embryonic stem cell derived-cardiomyocytes

**Figure 3:**
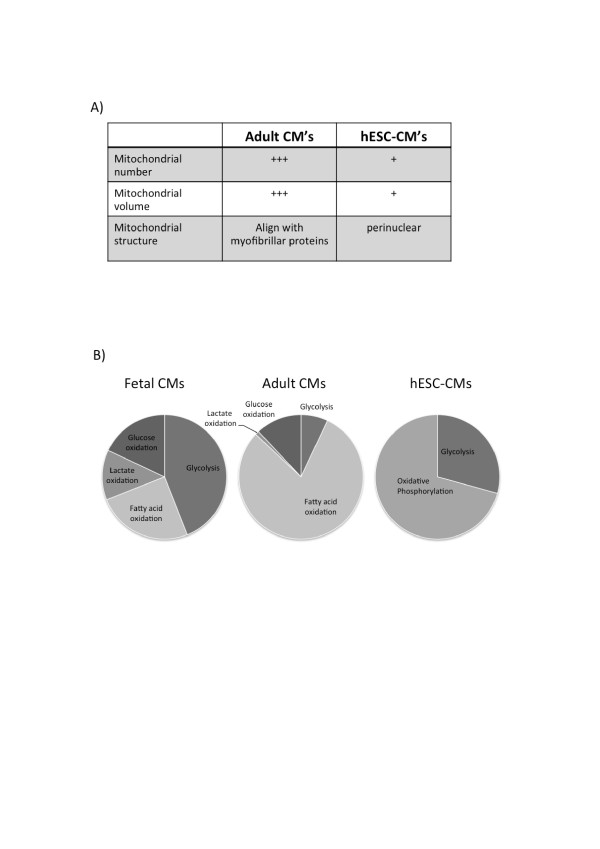
**Immature properties of mitochondrial bioenergetics in human embryonic stem cell-derived cardiomyocytes. (A)** Summary of differences in mitochondrial properties between adult cardiomyocytes (CMs) and human embryonic stem cell-derived CMs (hESC-CMs). **(B)** Relative contribution of glycolysis and oxidative metabolism in ATP production in fetal CMs, adult CMs and hESC-CMs.

Mature adult CMs have a mitochondrial volume that comprises over 35% of total cell volume [39,40]. Mitochondria in these cells are aligned with myofibrillar proteins such as sarcomeric α-actinin to form functional energetic units that facilitate energy production and excitation-contraction coupling during myocardial contraction [41,42]. In hESC-CMs, however, mitochondrial numbers are lower [43]. Mitochondria in these cells are also not aligned with myofibrillar proteins or sarcomeres [15] but are concentrated around the peri-nuclear area [44] (Figure 3A). The mitochondrial dynamic proteins DRP-1 and OPA1 in ESC-derived CMs are also expressed at a level that is considerably lower than in adult CMs [45].

Adult CMs derive their energy primarily from oxidative metabolism, with 90% of their acetyl-CoA from oxidation of fatty acid [46]. In contrast, embryonic and fetal CMs derive their ATP primarily through glycolysis, with oxidative metabolism of energy substrate accounting for <15% of the total acetyl-CoA derived from energy substrates [47]. Similarly, hESC-CMs are predominantly glycolytic, highlighting their immature and fetal like phenotypes [48], although oxidative metabolism [43], mainly in the form of lactate oxidation, is present [49] (Figure 3B). hESC-CMs express oxidative phosphorylation genes, although at low levels compared to fetal CMs [50].

## Developmental cues for maturation in human pluripotent stem cell derived-cardiomyocytes

### Thyroid hormones

One type of hormone that plays an important role during cardiac development and metabolism is thyroid hormone. Thyroid hormones regulate metabolism and gene transcription principally through binding of triiodothyronine (T3) to nuclear receptors, encoded by the *TRα* and *TRβ* genes. In rodent, T3 levels are low during the fetal period, and increase sharply shortly after birth, while in humans T3 remains low until approximately 30 weeks of gestation, but increases by over three-fold at term. Thyroid hormone has been shown to exert effects during different stages of cardiac development; it mediates a myosin heavy chain isoform switch from predominantly β in the fetal stage to α in the adult stage in mice [51,52]. However, since MHCβ is the predominant isoform in human, this isoform switch may not translate into functional and contractile changes [53]. Thyroid hormone has also been shown to regulate cardiac function by regulating the transcription of various cardiac genes [54]. The SR proteins Ca^2+^ ATPase [55] and phospholamban [56] have been shown to be upregulated by thyroid hormone. Other sarcolemmal ion channels, including Na^+^/K^+^ ATPase, NCX [57], and potassium channels, including Kv1.5, Kv4.2, and Kv4.3 [58], have also been shown to be regulated by thyroid hormones. Thyroid hormone also increases adrenergic activity by increasing β-adrenergic receptors, guanine-nucleotide regulatory proteins, and adenylyl cyclases, which can also affect SR function [59,60]. These changes may account for increased adrenergic stimulation in hyperthyroid patients; however, their effect during cardiac development is poorly understood. T3 supplementation during cardiac differentiation improves cardiac maturation in murine ESCs [61]. SR protein expression, including expression of calcium ATPase-2a and RyR-2, have been shown to be increased. NCX expression has also been shown to increase. Caffeine-induced calcium transient upstroke velocity as well as amplitude is also increased with T3 supplementation.

Our laboratory also demonstrated that T3 supplementation after cardiac differentiation increases cell volume, and promotes cell elongation. Action potential duration also decreases with T3 treatment post-differentiation, suggesting that T3 can enhance maturation both morphologically and electrophysiologically (WK and RAL, unpublished data).

### Adrenergic system

Besides thyroid hormone, adrenergic stimulation has also been shown to be important in the growth and maturation of the heart. During the first 2 postnatal weeks, both catecholamine and thyroid hormone levels increase significantly [62]. Adrenergic stimulation has been shown to mediate both the hypertrophic response as well as beating rates during postnatal development. Indeed, norepinephrine stimulates hypertrophy without hyperplasia by increasing cell volume, surface area, and intracellular protein content [63,64]. Both α-adrenergic and β-adrenergic stimulation has been shown to mediate maturation. α-Adrenoceptor stimulation has been shown to increase cell mass and L-type calcium channel currents [65]. The potassium current has also been shown to decrease with α-adrenergic stimulation, leading to increased myocardial contractility [66]. β-Adrenergic stimulation causes an increase in the beating rate in CMs [64]. In fetal and neonatal heart, however, β2-adrenoceptor predominates, which leads to both lusitropic and ionotropic responses [67]. Microarray analysis [68] shows that while β-1 adrenoceptors are expressed at extremely low levels in hESC-CMs, as is the case in human fetal hearts, β2 receptors are more abundantly expressed, although at levels still several hundred-fold less than in fetal hearts. However, this does suggest that hESC-CMs may be more responsive to β2 receptor stimulation. Indeed, various studies show that hESC-CMs respond to isoproterenol stimulation, leading to increased beating rate and decreases in magnitude of contraction, suggesting that the decrease in myofilament sensitivity to Ca^2+^ is greater than the increase in cytosolic Ca^2+^, even in late-stage cells [28,69].

## Transcriptomic, miR and epigenetic cues

Genetic and epigenetic analysis of hESC-derived CMs reveals a number of targets that may be important for cardiac maturation. These include a number of genes and microRNAs (miRNAs) as well as a number of histone modifications; however, the functional significance of these reported changes is only beginning to be unraveled.

### Transcriptome

Microarray analysis has revealed the upregulation of genes involved in cell communication and signal transduction as well as host defense responses in adult and fetal CMs but not in hESC-CMs [70]. In contrast, hESC-CMs show overexpression of genes involved in the process of cell development, highlighting the developing processes that hESCs are going through. Several genes that show increased expression from fetal to adult CMs are even less abundantly expressed in hESC-CMs, suggesting they can be used as maturation markers. These genes include the myosin genes *MYL2*, *MYL7*, *MYL3*, and *MYL11* as well as the cardiac troponin genes *TNNI3*, *TNNC1*, and *TNNT2*[70,71]. Calcium handling genes have also been shown to be upregulated in hESC-CM clusters [71]. In one study, Beqqali and colleagues [72] identified a number of novel genes related to cardiac differentiation in hESC-CMs, including *SRD5A2L2*, *SYNPO2L*, *THC2339346*, *THC1564329*, and *THC1452070*. These genes were expressed in human fetal CMs, suggesting a role in cardiac development.

hESC-CMs possess a transcriptomic pattern that is unique. Upregulation of genes exclusively in hESC-CMs may serve as important cues for cardiac maturation *in vitro* as opposed to the *in vivo* maturation process from fetal to adult CMs [70]. Pathway analysis of microarray data also reveals novel pathways that are important in the cardiogenesis process *in vitro*. Genes in the focal adhesion pathway have been shown to be upregulated in hESC-CMs [71]. These genes have been implicated in a diverse number of cellular processes, including tissue remodeling, cell migration, embryogenesis, growth factor signaling, cell cycle progression, and cell survival. One novel pathway discovered by Xu and colleagues [70] involves the transcriptional factor network that links to the peroxisome proliferator-activated receptor (PPAR) signaling pathway. The activation of PPARD in this signaling pathway involves the binding of prostaglandin I_2_[3].

### MicroRNA

miRNAs are a class of evolutionarily conserved small (20 to 26 nucleotides in length) non-protein-coding RNAs that negatively regulate gene expression by affecting mRNA stability and translation [73]. They play important roles in the post-transcriptional regulation of gene expression, and recent studies have established critical functions for these miRNAs in cardiac development [74].

miR-1 is the most abundant miRNA in the mammalian heart. It is clustered together with another miRNA, miR-133, both of which have been found to be essential for cardiac development. The expression of muscle contractile proteins is tightly regulated during cardiac development and maturation. The expression of muscle-specific myosin genes is regulated by a group of intronic miRNAs, including miR-208a, miR-208b and miR-499, which are embedded within the introns of *Myh6*, *Myh7* and *Myh7b*, respectively [74].

Although several clusters of miRNA are important for cardiac development and maturation, only miR-1, miR-133 and miR-499 are significantly induced during cardiac differentiation in hESCs [75-78]. Moreover, miR-1, miR-144 and miR-499 are the most differentially expressed miRNAs between hESCs, hESC-CMs, human fetal CMs and human adult CMs [77]. The expression of these miRNAs has since been manipulated to facilitate maturation of hESC-CMs. When overexpressed during pre-cardiac differentiation, miR-1 induces expression of cardiac marker genes in both mouse and human ESCs [78] and EBs [76,77]. Post-differentiation, miR-1 overexpression in hESC-CMs did not change the expression of cardiac contractile proteins, including α-MHC and β-MHC, MLC2V, α-actinin and troponin T [77]. However, miR-1 overexpression did promote electrophysiological maturation with a decrease in action potential duration and a more hyperpolarized resting membrane potential. This was accompanied by upregulation of Kir2.1, Kv1.4, HERG and DHPR and downregulation of HCN4. miR-1 overexpression also results in maturation of calcium handling in hESC-CMs, increasing calcium transient amplitude and upstroke velocity, which is accompanied by increased expression of junctin (Jnct), triadin (Trdn) and ryanodine (RyR2) mRNA.

Overexpression of miR-499 in human cardiac progenitor cells [75] and hESCs induces expression of cardiac gene markers, including β-MHC [77]. In hESC-CMs the percentage of ventricular CMs in EBs overexpressing miR-499 increases significantly [77]. hESC-CMs overexpressing miR-499 show increases in cardiac contractile proteins, including α-MHC and β-MHC, MLC2V, α-actinin and troponin T [77]. Overexpression of miR-499 does not induce changes in calcium handling in hESC-CMs that are characteristic of more mature ventricular CMs [77]. Therefore, while both miR-1 and miR-499 appear to be potent inducers of cardiomyogenic differentiation of stem cells, miR-499 promotes ventricular specificity after initiation of cardiac differentiation while miR-1 induces a more mature ventricular CM phenotype than miR-499 [77].

### Histone modification

Besides miRNAs, chromatin modifications and epigenetic changes are central to regulation of gene expression [79,80]. Increased acetylation of amino-terminal lysine residues of histones H3 and H4 by histone acetylases correlates with increased transcription, as the folded chromatin becomes more accessible to transcriptional machinery [81]. On a more genome-wide level, important chromatin patterns of the embryonic epigenetic landscape have been identified, with a balance between active (H3K4me3-enriched) and silent (H3K27me3-enriched) transcription maintained by specific histone methyltransferases [82].

Increased H3 acetylation in hESCs/human iPSC-CMs has been shown in three-dimensional cultures and with the administration of the histone deacetylase inhibitor trichostatin A. This increase in H3 acetylation is accompanied by augmented expression of cardiac genes, including those encoding α-MHC, ERG1b and KCNQ1. Moreover, the increase in H3 acetylation induces a more mature electrophysiological profile in hESC-CMs, which enhances their responses to IKr inhibitors E4031, nifekalant and sotalol [83].

Our group has shown that levels of H3K4me3 were specifically enriched on cardiac gene promotors that regulate *MLC2V*, *MLC2A*, *cTNT* and *ANP* gene expression, as well as calcium handling genes encoding PLN, DHPR, ASPH, TRDN, and other ion channel proteins such as SCN5A and KCNA4 in hESC-CMs. This lysine trimethylation can be further enhanced by the histone deacetylase inhibitor valproic acid [84]. Indeed, valproic acid in hESC-CMs induces expression of β-MHC protein as well as ANP, and leads to an increase in cell size, consistent with the induction of hypertrophy observed in fetal CMs [85].

## Physical cues

In addition to biological cues for maturation, CMs in their natural environment also possess physical properties, including topographical cues, that induce changes in cell morphology, as well as electrical and physical properties [86]. Mature adult CMs in native heart are aligned in a highly organized manner and can support fast action potential conduction that is anisotropic with distinct transverse and longitudinal velocities to support generation of high contractile stresses [87,88]. In contrast, hPSC-CMs cultured as monolayers exhibited contractile stresses and conduction velocities that are an order of magnitude lower than those in adult human myocardium [89].

Fabricated cell culture substrates that mimic the native environment found in the heart may improve the functional maturation of hPSC-CMs. Indeed, microtopographical cues have been shown to be a stronger determinant of cell orientation than electrical stimulation [90]. hESC-CMs cultured on wrinkled substrate with nano to micro topographies show alignment and display organized sarcomeric structures with banding, and alignment of connexin-43 proteins near cell-cell junctions [91,92]. Our group has shown that an aligned monolayer of hESC-CMs grown on shrink film configurable multiscale wrinkled substrate also exhibits an anisotropic propagation with faster longitudinal conduction velocity parallel to the direction of wrinkles than that of the transverse conduction velocity [92]. The aligned anisotropic hESC-CMs are more resistant to re-entrant arrhythmia [93]. Human iPSC-CMs cultured on similar aligned micro-grooved substrate also have improved sarcomeric structure. Calcium cycling properties show maturation with a decrease in upstroke velocity as well as caffeine-induced calcium release when compared to control monolayers [94].

Culturing of hPSC-CMs in three-dimensional cardiac tissue patches improves alignment and electrical conduction with a significant increase in action potential velocity and contractile force [95]. Unlike two-dimensional constructs, three-dimensional culturing of hESC-CMs requires the addition of stromal cells to improve their survival and alignment [95]. Similar three-dimensional cultures with induced pluripotent stem cardiac progenitor cells also show differentiation into CMs with improvement of alignment and expression of gap-junctions and adherent molecules at cell-cell junctions and improved electrical conduction [96].

## Facilitated maturation of human pluripotent stem cell derived-cardiomyocytes

### Facilitated maturation of electrophysiological and calcium handling properties

Efforts to understand the biology and electrophysiology of hESC-CMs have led to the identification of a number of key differences between hESC-CMs and their adult mature counterpart. This has led to the development of different strategies to facilitate electrophysiological maturation in hESC-CMs. As previously described, expression of the calcium handling proteins calsequestrin and phospholamban is almost completely absent in hESC-CMs. Thus, one strategy for facilitating maturation of calcium handling in hESC-CMs is to induce forced expression of the ‘missing’ protein. Indeed, our laboratory has shown that forced expression of calsequestrin [36] leads to functional improvements of calcium transient parameters, with increased upstroke velocity and calcium transient amplitude. However, other electrical properties of these calsequestrin overexpressing hESC-CMs remain immature.

Expression of the inward rectifying potassium channel Kir2.1 is absent in hESC-CMs and is the key determinant of their immature electrophysiological profile [97]. Forced Kir2.1 expression alone sufficed to render the electrical phenotype indistinguishable from that of primary adult ventricular cells [20,97]. However, these cells continue to exhibit immature calcium handling properties, with a small calcium transient amplitude as well as a slow upstroke velocity.

Endogenous pacing in neonatal CMs promotes maturation of both electrical and calcium handling properties. In view of this, we have subjected hESC-CMs, which would otherwise spontaneously beat in a weak unsustained and sporadic manner, to field-stimulation to induce forced electrical pacing. Electrical conditioning robustly led to many aspects of cellular maturation of hESC-CMs, including electrophysiological maturation without phase 4-depolarization similar to Kir2.1 gene transfer, Ca^2+^-handing maturation with increased peak Ca^2+^ transient amplitude and SR Ca^2+^ load, and structured organization of myofilaments, as well as upregulation of contractile and t-tubule biogenesis proteins [97].

### Facilitated maturation of metabolic properties

As previously discussed, while hESC-CMs are metabolically active, they possess an immature metabolic profile that is predominantly glycolytic. In normal cardiac development, metabolic or mitochondrial maturation does not occur until after birth, when there is an increase in contractile function, exposure to fatty acid as a substrate for energy, and elevated oxygen levels. Using a combination of β-adrenergic stimulation (isoproterenol) and fatty acid supplementation to mimic post-natal developmental processes, our laboratory was able to increase mitochondrial energetics. These supplements increased mitochondrial volume as well as mitochondrial membrane potential of the cells. Tricarboxylic acid cycle enzyme activity was also increased (WK and RAL, unpublished data). These results are consistent with other reports that shifted energy metabolism of human iPSC-CMs from glycolytic to predominantly oxidative through the use of galactose. Galactose alone as well as in combination with fatty acids shifts energy metabolism from predominantly glycolytic to oxidative. Mitochondrial reserve capacity and maximum mitochondrial capacity are also increased with galactose and fatty acid supplementation [98,99]. While there is no change in mRNA expression of key metabolic genes, expression of enzymes of the electron transport chain complexes I to IV is significantly increased in galactose and fatty acid supplemented cells; however, the levels of expression are still significantly lower than in adult CMs [98].

## Conclusion

hESC-CMs provide an excellent source of cells for myocardial repair and regeneration, although the differences between them and mature CMs have limited their effectiveness for regeneration and cell replacement therapy. With better understanding of the developmental cues leading to maturation of hPSC-CMs, as well as recent advances in fabrication of two-dimensional and three-dimensional culture substrates, strategies to facilitate maturation of these cells can be developed. This would enable the use of both hESC-CMs and human iPSC-CMs as safe and efficient sources for cell and tissue replacement therapy for the treatment of heart disease.

## Note

This article is part of a thematic series on *Cardiovascular regeneration* edited by Ronald Li. Other articles in the series can be found online at http://stemcellres.com/series/cardiovascular.

## Abbreviations

BMP: Bone morphogenetic protein; CM: Cardiomyocyte; EB: Embryoid body; hESC: human embryonic stem cell; hPSC: Human pluripotent stem cell; iPSC: Induced pluripotent stem cell; miRNA: microRNA; NCX: Sodium calcium ion exchanger; PPAR: Peroxisome proliferator-activated receptor; RyR: Ryanodine receptor; SERCA: Sarco/endoplasmic reticulum Ca^2+^ ATPase; SR: Sarcoplasmic reticulum; T3: Triiodothyronine.

## Competing interests

The authors declare that they have no competing interests.
